# Establishment of a strain of haemophilia-A pigs by xenografting of foetal testicular tissue from neonatally moribund cloned pigs

**DOI:** 10.1038/s41598-017-17017-6

**Published:** 2017-12-05

**Authors:** Hiroyuki Kaneko, Kazuhiro Kikuchi, Michiko Nakai, Daiichiro Fuchimoto, Shunichi Suzuki, Shoichiro Sembon, Junko Noguchi, Akira Onishi

**Affiliations:** 1 Institute of Agrobiological Sciences, National Agriculture and Food Research Organization (NARO), Tsukuba, Ibaraki 305-8602 Japan; 2 The United Graduate School of Veterinary Science, Yamaguchi University, Yoshida, Yamaguchi 753-8515 Japan; 3 Nihon University, College of Bioresource Sciences, Fujisawa, Kanagawa 252-0880 Japan; 4 Present Address: NARO, Tsukuba, Ibaraki, 305-8517 Japan

## Abstract

Grafting of testicular tissue into immunodeficient mice makes it possible to obtain functional sperm from immature donor animals that cannot be used for reproduction. We have developed a porcine model of human haemophilia A (haemophilia-A pigs) by nuclear transfer cloning from foetal fibroblasts after disruption of the X-linked coagulation factor VIII (F8) gene. Despite having a recessive condition, female F8^+/−^ cloned pigs died of severe bleeding at an early age, as was the case for male F8^−/Y^ cloned pigs, thus making it impossible to obtain progeny. In this study, therefore, we produced sperm from F8^−/Y^ cloned pigs by grafting their foetal testicular tissue into nude mice. Two F8^+/−^ female pigs were generated from oocytes injected with xenogeneic sperm. Unlike the F8^+/−^ cloned pigs, they remained asymptomatic, and delivered five F8^−/Y^ and four F8^+/−^ pigs after being crossed with wild-type boars. The descendant F8^−/Y^ pigs conserved the haemophilia phenotype. Thus, the present F8^+/−^ pigs show resolution of the phenotypic abnormality, and will facilitate production of F8^−/Y^ pigs as founders of a strain of haemophilia-A pigs for the development of new therapeutics for haemophilia A. This strategy will be applicable to other genetically modified pigs.

## Introduction

Grafting of testicular tissue into immunodeficient mice is a promising way of harvesting sperm from different donor species that have not yet reached sexual maturation. A major application of testicular xenografting is the salvage of genetic information from valuable immature animals by generating offspring using their xenogeneic sperm. Since the technique was first described by Honaramooz *et al*.^1^, testicular tissues prepared from neonatal or young donors of many species have been tested for recovery of sperm. Reports published so far have indicated that neonatal donor testicular tissues after cryopreservation have the ability to initiate spermatogenesis in host mice: their germ cells reached the spermatocyte (human^2,3^ and primate^4^), spermatid (pig^5,6^ and sheep^7^) or sperm (rabbit^8^ and pig^9,10^). The ability of xenogeneic sperm to generate offspring has been demonstrated by intracytoplasmic sperm injection (ICSI) using sperm from cryopreserved testicular tissues of neonatal rabbits^8^ and pigs^10,11^. Furthermore, the reproductive ability of offspring produced using such xenogeneic sperm has also been confirmed in pigs^12^. Thus, testicular xenografting combined with cryopreservation makes it possible to generate offspring from neonatal testicular tissue after long-term cryopreservation. A recent study has indicated that porcine foetal testicular tissue, even when collected in the early gestational period, acquires the capacity to produce sperm after xenografting^13^. However, it remains to be determined whether xenogeneic sperm derived from foetal testis have full developmental competence.

Pigs closely resemble humans in terms of anatomy, physiology and pathology. They are much longer lived than mice and rats, thus allowing long-term evaluation of new medicines and medical procedures based on observation of individual animals. Therefore, genetically modified pigs have been gaining increased attention as possible models of human disease in preclinical studies^14^.

Haemophilia-A is an X-linked recessive disorder in humans caused by mutations in the coagulation factor VIII (F8) gene, resulting in dysfunction of blood coagulation^15^. Hemizygous (F8^−/Y^) men exhibit the severe bleeding phenotype, characterized by features such as spontaneous hemorrhage in the joints and muscles, whereas heterozygous women (F8^+/−^) are usually asymptomatic carriers^15,16^. We have developed a porcine model of human haemophilia-A (the haemophilia-A pig) by nuclear transfer cloning from male foetal fibroblasts harboring targeted disruption of the F8 gene^17^. The resulting F8^−/Y^ cloned pigs exhibited ecchymoses in the cheek, forelimbs and hind limbs due to extreme paucity of F8 clotting activity and died within a few days after birth^17^. In a subsequent study, we attempted production of female F8-targeted pigs by cloning from female foetal fibroblasts after disruption of the F8 gene (Supplementary Methods S1), as it was expected that these pigs would survive and deliver progeny harboring the disrupted F8 gene after crossing with wild-type boars. We produced 7 female F8-targeted pigs, but all of the cloned pigs died of bleeding at an early age (Supplementary Fig. S1), even though they possessed one wild-type F8 allele (F8^+/−^) (Supplementary Fig. S2). This unforeseen obstacle made it impossible to obtain progeny from the haemophilia-A pigs. One possible way of overcoming this problem would be to generate F8^+/−^ offspring using sperm obtained from immature F8^−/Y^ cloned pigs by testicular xenografting. If the bleeding phenotype in the F8^+/−^ cloned pigs were associated with epigenetic modification, such trials might be justified as, in cloned mice, it has been found that when pups were produced using sperm collected from testicular tissue that had been grafted into nude mice, the phenotypic abnormality caused by epigenetic errors was not transferred to their progeny^18^.

**Figure 1:**
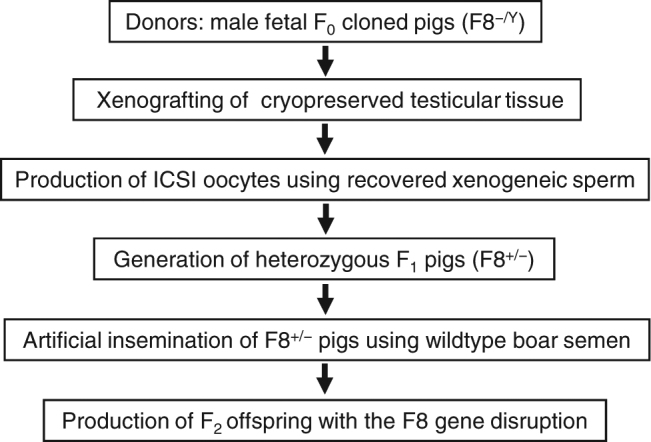
Experimental design. The critical point of the present study was whether asymptomatic F8^+/−^ pigs could be produced by ICSI using sperm derived from foetal F8^−/Y^ pigs.

In the present study, therefore, we cryopreserved testicular tissue of F8^−/Y^ cloned pigs (defined as the F_0_ generation) and grafted them into nude mice. To avoid sudden death of the neonatal F8^−/Y^ pigs due to accidental or spontaneous bleeding, testicular tissues were collected from F8^−/Y^ embryos in the mid- to late gestational period. We then injected sperm recovered from the host mice into *in vitro*-matured oocytes of wild-type gilts (F8^+/+^) to generate offspring. After assessment of the genotype and phenotype of the offspring (F_1_), we bred F8^+/−^ pigs as founders with wild-type boars (F8^+/Y^) and attempted to isolate the F8^−/Y^ phenotype at the F_2_ generation (Fig. 1).

## Results

### Production of foetal F8^**−**/Y^ cloned pigs (F_0_ generation)

**Figure 2:**
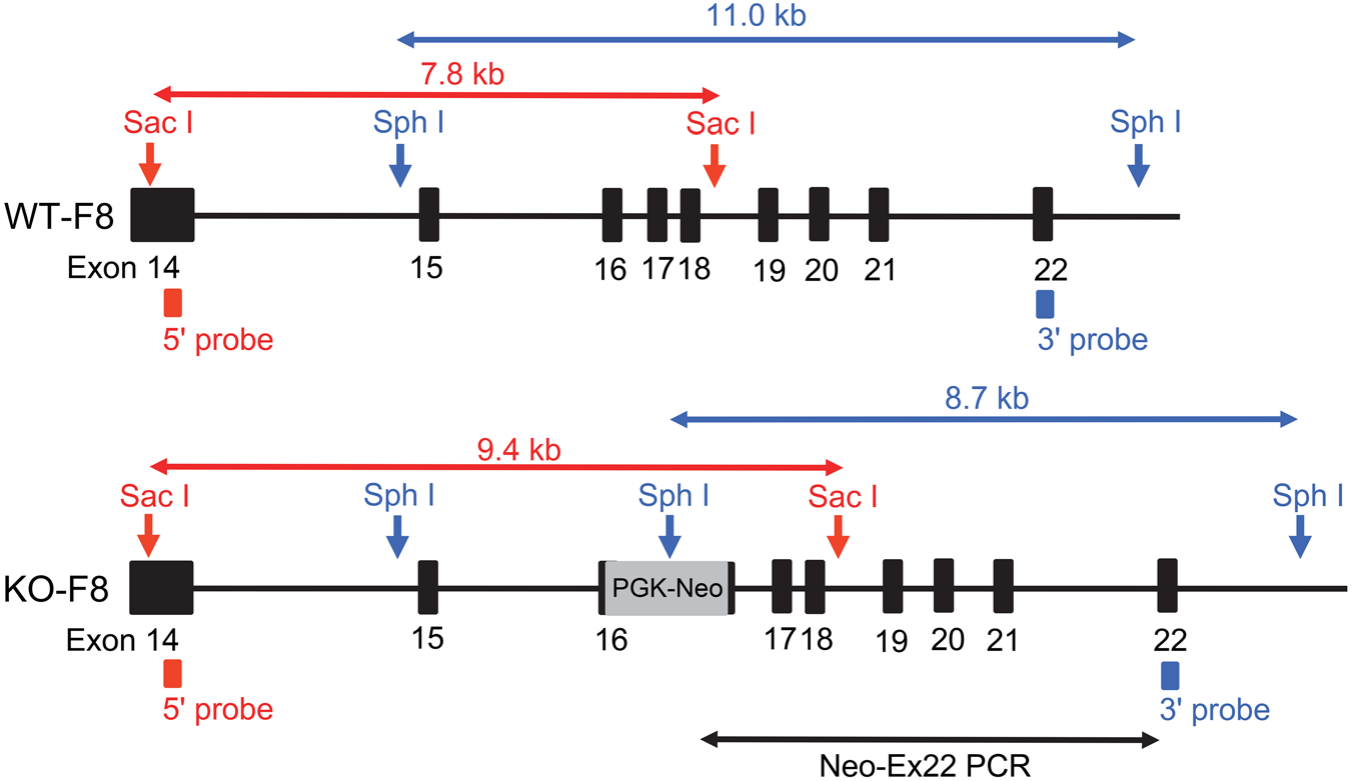
Diagram of part of the wild-type porcine F8 (WT-F8) and the targeted F8 (KO-F8) genes. PGK and the neomycin resistance gene (PGK-Neo) with deletion of 11 nucleotides by targeting was inserted into exon 16 of the targeted F8 gene. The position of the PCR primer and amplified DNA fragment is indicated by a black arrow (Neo-Ex22 PCR). The positions of the Southern blotting probes are indicated by red (5′ probe) and blue (3′ probe) columns. DNA fragments of Sac I-digested genomic DNA detected with the 5′ (Ex14) probe and Sph I-digested DNA detected with the 3′ (Ex22) probe are indicated by red and blue arrows, respectively (modified from Kashiwakura *et al*.^17^).

**Figure 3:**
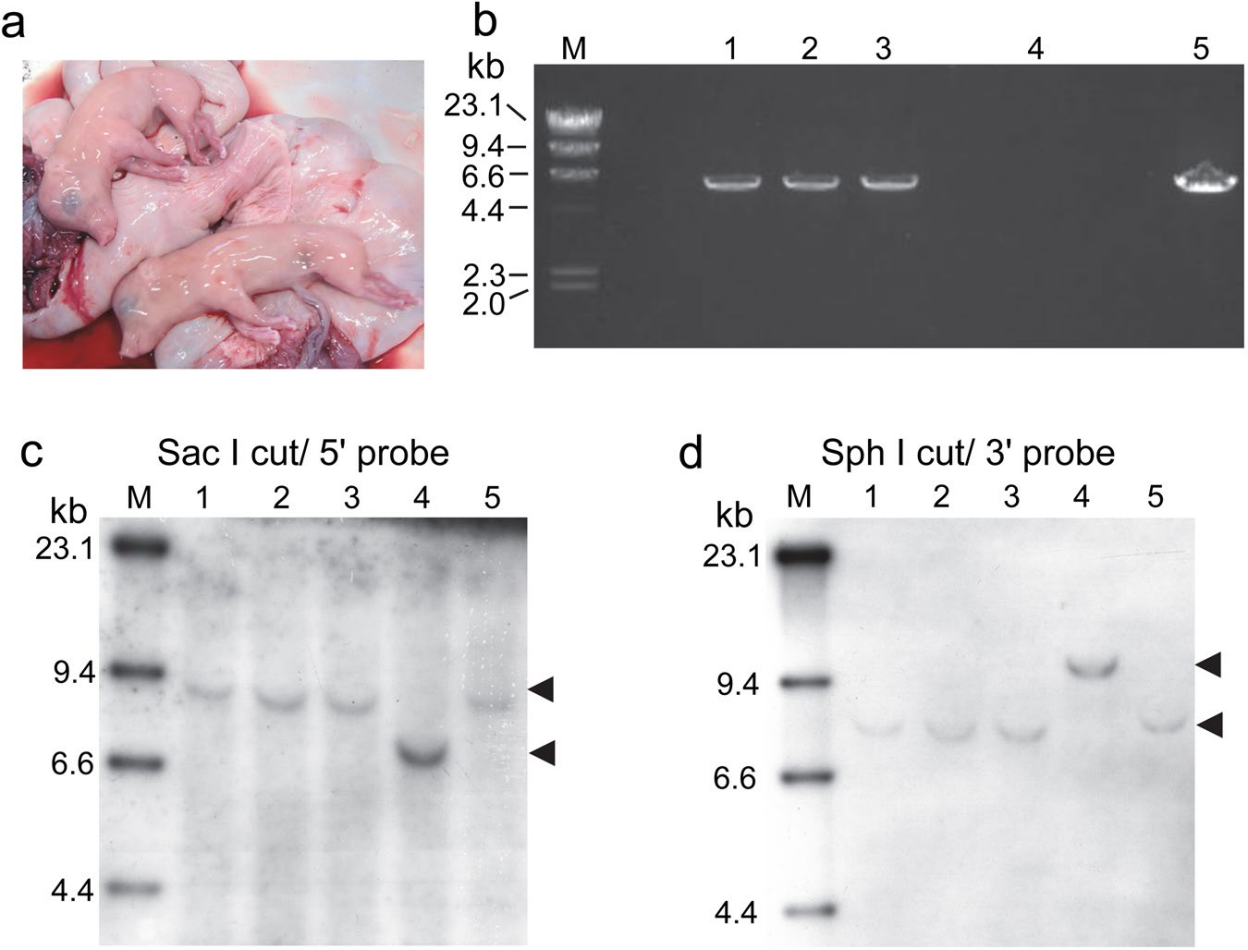
Production of foetal F8^−/Y^ cloned pigs. (**a**) Two foetal cloned pigs obtained at 69 days after embryo transfer. (**b**) Amplification of the F8-targeted allele by Neo-Ex22 PCR from genomic DNA extracted from the three foetal cloned pigs. Lanes 1, 2 and 3, foetal cloned pigs; lane 4, wild-type control; Lane 5, positive control, a male F8^−/Y^ pig produced in the previous study^17^. Southern blotting of (**c**) Sac I- and (**d**) Sph I-digested genomic DNA of three foetal cloned pigs. Lanes 1, 2 and 3, foetal cloned pigs; lane 4, wild-type control; Lane 5, positive control, a male F8^−/Y^ pig produced in the previous study^17^. Arrowheads indicate hybridized signals. M indicates molecular weight marker (λ-Hind III). The images of PCR (Fig. 3b) and blots (Fig. 3c and d) have been cropped (uncropped images are shown in Supplementary Fig. S3).

Firstly, we inactivated the F8 gene in male porcine embryonic fibroblasts with the F8-targeting vector and produced embryos by nuclear transfer of the F8-targeted cells (Fig. 2). After transfer of the cloned embryos to the oviducts of 25 recipient pigs, two recipients conceived; two cloned foetuses were collected at 69 days after embryo transfer and one at 83 days (Fig. 3a). Neo-Ex22 PCR (Fig. 2) amplified a 6.0-kb DNA fragment from the genomic DNA of these foetuses (Fig. 3b). Southern blotting of the Sac I-digested DNA using a 5′ probe (Fig. 2) detected upper mobility shifts of bands from the three cloned foetuses, as compared to the band mobility for wild-type pig (Fig. 3c), and a 3′ probe on the Sph I-digested DNA recognized a lower shift of bands in these foetuses (Fig. 3d). These analyses confirmed that the 3 foetal cloned pigs were hemizygous (F8^−/Y^). In addition, quantitative RT-PCR confirmed that F8 mRNA was not detectable in the liver of these foetuses.

### Development of testicular tissue from foetal F8^**−**/Y^ cloned pigs in host mice

**Figure 4:**
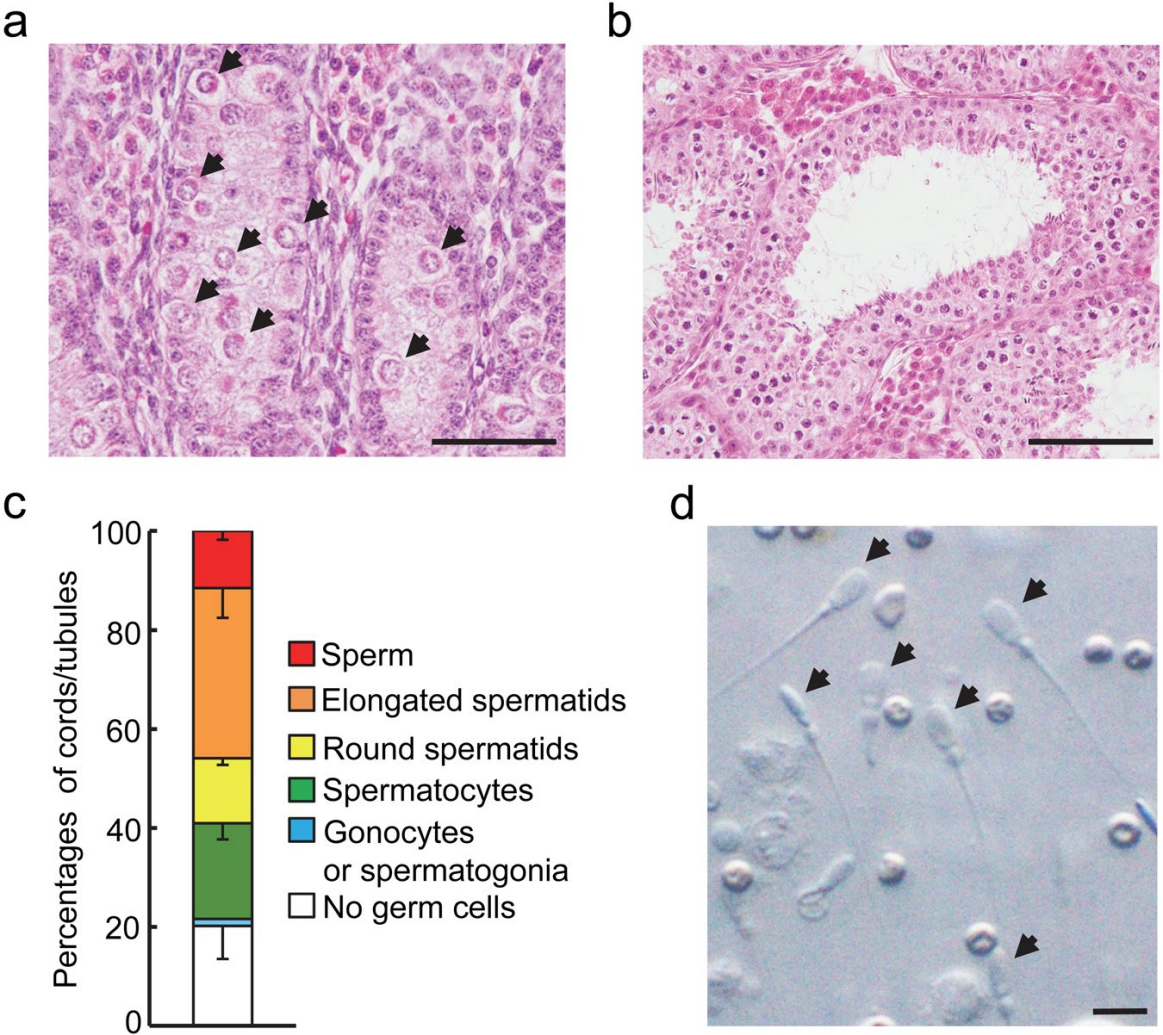
Growth and differentiation of testicular tissue from foetal F8^−/Y^ cloned pigs in host mice. Representative features of (**a**) testes of foetal donors (F8^−/Y^ pigs) between 69 and 83 days after embryo transfer and (**b**) porcine testicular tissue obtained from host mice between 300 and 369 days after grafting. (**c**) Mean percentages (±SEM) of seminiferous cord or tubule cross-sections in the grafted tissue obtained from 4 mice, as classified by the most advanced type of germ cell present. (**d**) Sperm retrieved from xenografts. Scale bars represent 50 µm in image (**a**), 100 µm in (**b**) and 10 µm in (**d**). Arrows indicate gonocytes (**a**) and sperm (**d**), respectively.

Before xenografting, testes of foetal F8^−/Y^ pigs contained seminiferous cords consisting of only gonocytes and Sertoli cells (Fig. 4a). Four out of 7 castrated mice survived after receiving cryopreserved testicular fragments. At graft recovery between 300 and 369 days post-grafting, the weights of the visible grafts per mouse reached 3.9 ± 0.4 (mean ± SEM, n = 4) g, being considerably increased from the weight of testicular fragments before grafting (16 mg). The grafted tissue developed seminiferous tubules containing elongated spermatids and sperm (Fig. 4b): the percentage of tubules containing sperm or elongated spermatids was 11.5 ± 1.7% (mean ± SEM) or 34.4 ± 6.0% (Fig. 4c). A number of sperm were recovered and many of them were morphologically normal (Fig. 4d). Serum concentrations of total inhibin and testosterone in the four host mice were 4.1 ± 0.3 (mean ± SEM) ng/ml and 0.6 ± 0.1 ng/ml, respectively: much higher (P < 0.01) than those in the five castrated male mice that had not received testicular grafts (inhibin, 0.3 ± 0.1 ng/ml; testosterone, 0.04 ± 0.01 ng/ml).

### Generation of F_1_ offspring by ICSI using sperm derived from foetal F8^**−**/Y^ cloned pigs

**Figure 5:**
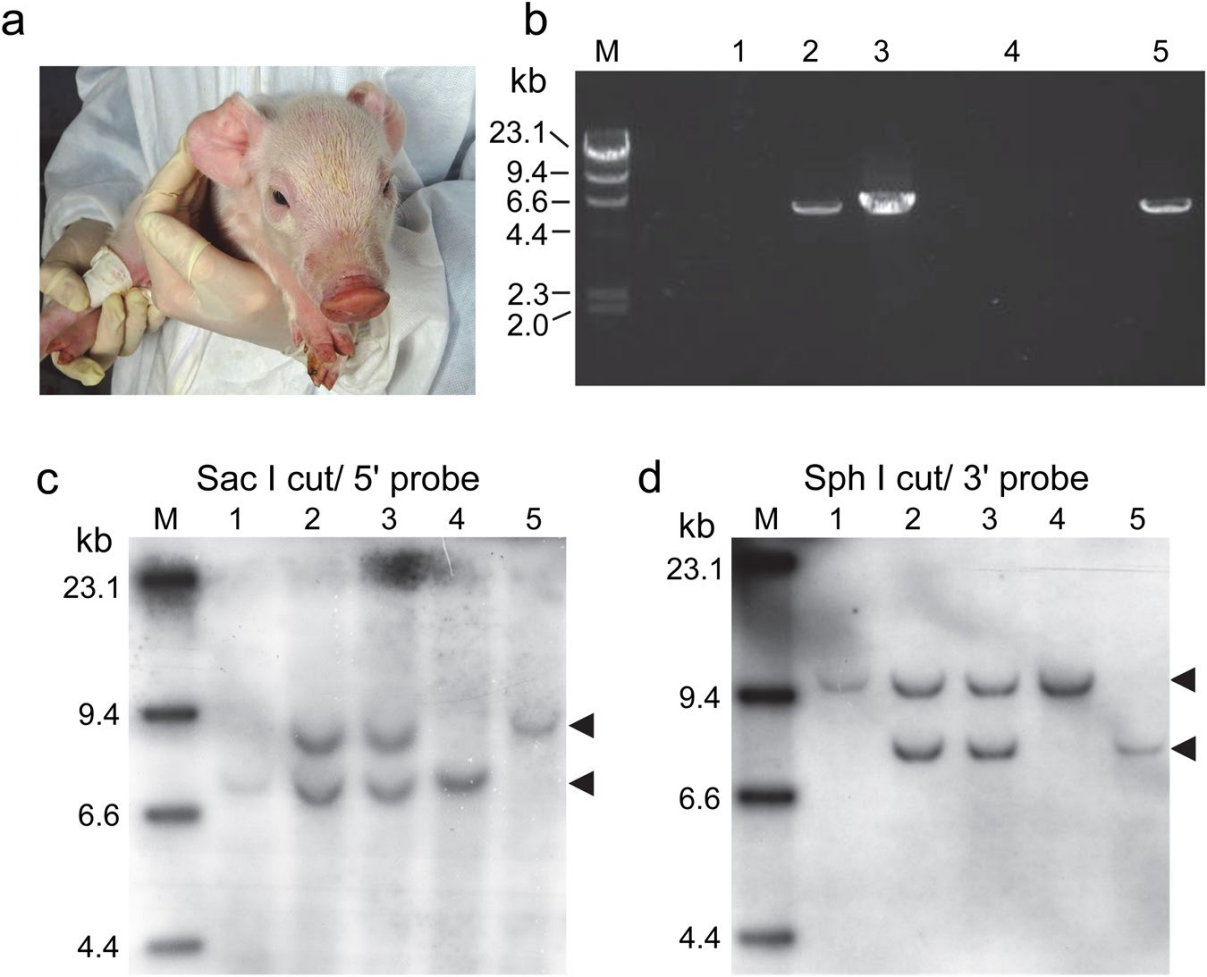
Generation of F_1_ offspring from immature testicular germ cells of foetal F8^−/Y^ pigs. (**a**) A female piglet on 1 day after birth, showing no bleeding tendency. (**b**) Amplification of the F8 targeted allele by Neo-Ex22 PCR using genomic DNA extracted from F_1_ offspring. Lane 1, male F_1_ piglet; lanes 2 and 3, female F_1_ piglets; lane 4, wild-type control; lane 5, positive control, foetal F8^−/Y^ pig produced in the present study. Southern blotting of (**c**) Sac I- and (**d**) Sph I-digested genomic DNA from F_1_ offspring. Lane 1, male F_1_ piglet; lanes 2 and 3, female F_1_ piglets; lane 4, wild-type control; lane 5, foetal F8^−/Y^ pig. Arrowheads indicate hybridized signals. M indicates molecular weight marker (λ-Hind III). The images of PCR (Fig. 5b) and blots (Fig. 5c and d) have been cropped (uncropped images are shown in Supplementary Fig. S4).

We injected sperm recovered from xenografts into wild-type porcine mature oocytes and then transferred the sperm-injected oocytes to the oviducts of 4 recipient gilts. Two of the gilts gave birth: one delivered 1 female (birth weight: 1.3 kg) and 1 male (1.7 kg) piglets 114 days after transfer of sperm-injected oocytes, and the other 1 female piglet (1.1 kg) at 118 days. These piglets did not show any bleeding tendency and survived (Fig. 5a). The two female piglets were found to be heterozygous (F8^+/−^), defined as having one copy of the targeted F8 and one copy of the wild-type F8 alleles as assessed by PCR (Fig. 5b) and Southern blotting (Fig. 5c and d). The targeted F8 allele was not detected in the male piglet (F8^+/Y^) (Fig. 5b–d). The activity of F8 in the plasma of the F8^+/−^ piglets, determined by the clotting time method for human F8, corresponded to 387% and 354% of the activity in normal human plasma, whereas the activity was 850% in the male wild-type piglet.

### Generation of F_2_ offspring harboring the F8 gene disruption

**Figure 6:**
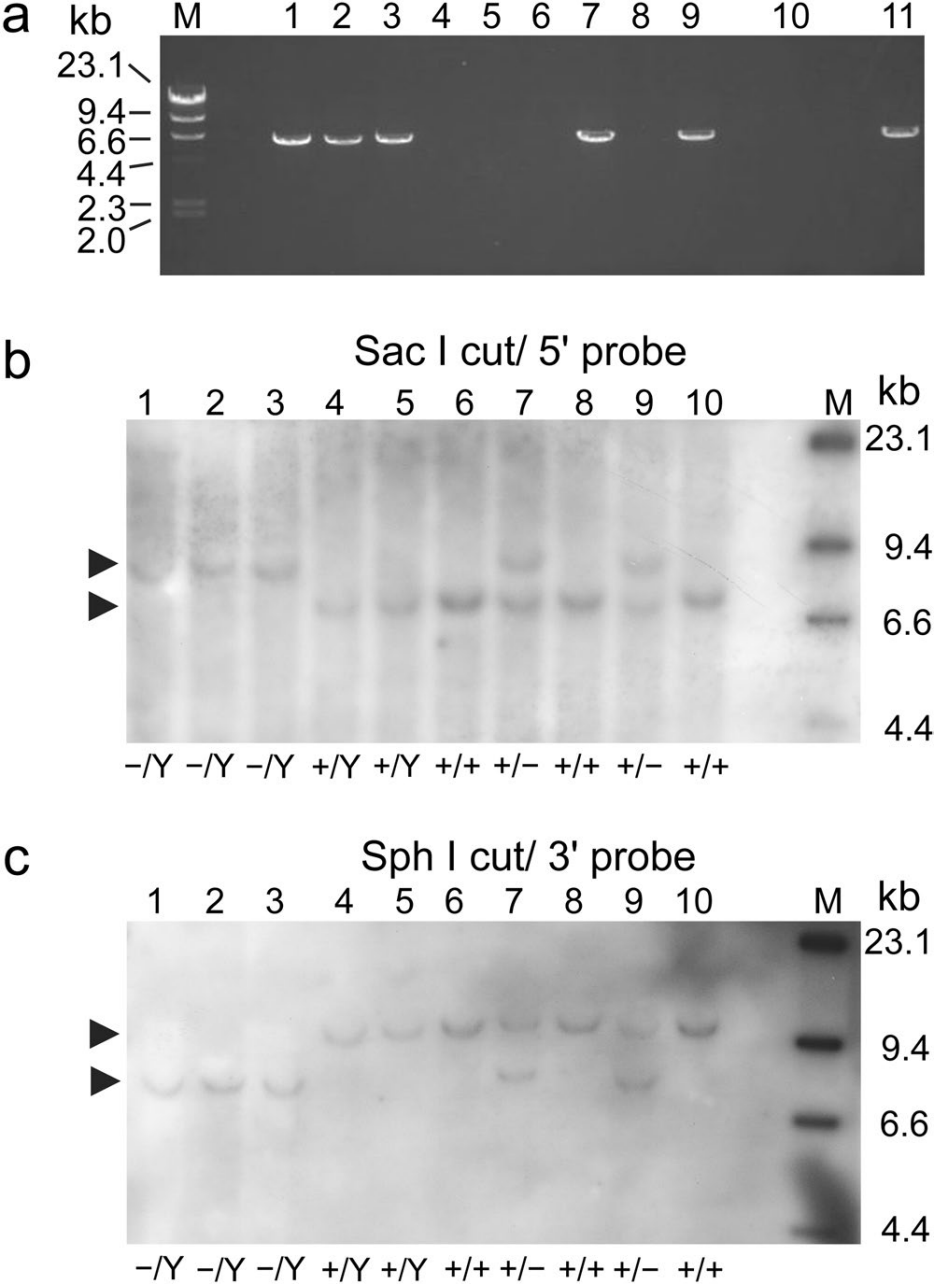
Representative genotyping of the F_2_ offspring. (**a**) Amplification of the F8-targeted allele by Neo-Ex22 PCR from genomic DNA extracted from a litter of F_2_ piglets (1st delivery from HY27-671, see Table 1). Lanes 1 to 5, male piglets; lanes 6 to 9, female piglets; lane 10, wild-type control; lane 11, positive control, foetal F8^−/Y^ pig produced in the present study. Southern blotting of (**b**) Sac I- and (**c**) Sph I-digested genomic DNA extracted from the same pigs. Lanes 1 to 5, male piglets; lanes 6 to 9, female piglets; lane 10, wild-type control. Arrowheads indicate hybridized signals. The resulting genotypes are indicated at the bottom of each image, and were consistent with those determined by PCR analysis shown in (**a**). M indicates molecular weight marker (λ-Hind III). The images of PCR (Fig. 6a) and blots (Fig. 6b and c) have been cropped (uncropped images are shown in Supplementary Fig. S5).

Two F8^+/−^ pigs had been used as founders and delivered a total of 17 piglets (9 males and 8 females) by 3 parturitions after being artificially inseminated with semen of wild-type (F8^+/Y^) boars (Landrace or Large White) (Table 1). From among the F_2_ offspring, 5 male piglets were found to be hemizygous (F8^−/Y^) by PCR (Fig. 6a) and Southern blotting (Fig. 6b and c): four female piglets were heterozygous (F8^+/−^) and the other 8 were wild-type piglets (four F8^+/Y^ and four F8^+/+^).

**Table 1:** Perinatal characteristics of F_2_ offspring delivered from F8^+/−^ pigs.

Founder^a^	Pregnancy^b^	No. of piglets^c^		No. of F8^−/Y^ piglets^d^	No. of F8^+/−^ piglets^d^	No. of F8^+/Y^ piglets^d^	No. of F8^+/+^ piglets^d^
		male	female				
HY27-671	1st (114)	5	4	3 (2)	2 (2)	2 (2)	2 (2)
	2nd (116)	1	2	1 (1)	2 (2)	0	0
HY28–85	1st (113)	3	2	1 (1)	0	2 (2)	2 (2)
		9	8	5 (4)	4 (4)	4 (4)	4 (4)
Weight^e^		1.2 ± 0.1	1.3 ± 0.2	1.2 ± 0.2	1.2 ± 0.2	1.2 ± 0.2	1.3 ± 0.2

^a^Two F8^+/−^ pigs produced in the present study were used.

^b^Each pregnancy followed by the duration of pregnancy (in parenthesis).

^c^Total number of piglets delivered.

^d^Number of F8^−/Y^, F8^+/−^, F8^+/Y^ or F8^+/+^ piglets delivered, followed by the number of live-born piglets (in parenthesis).

^e^Birth weight: Mean ± SEM.

### Analysis of phenotype in the F_2_ offspring

**Figure 7:**
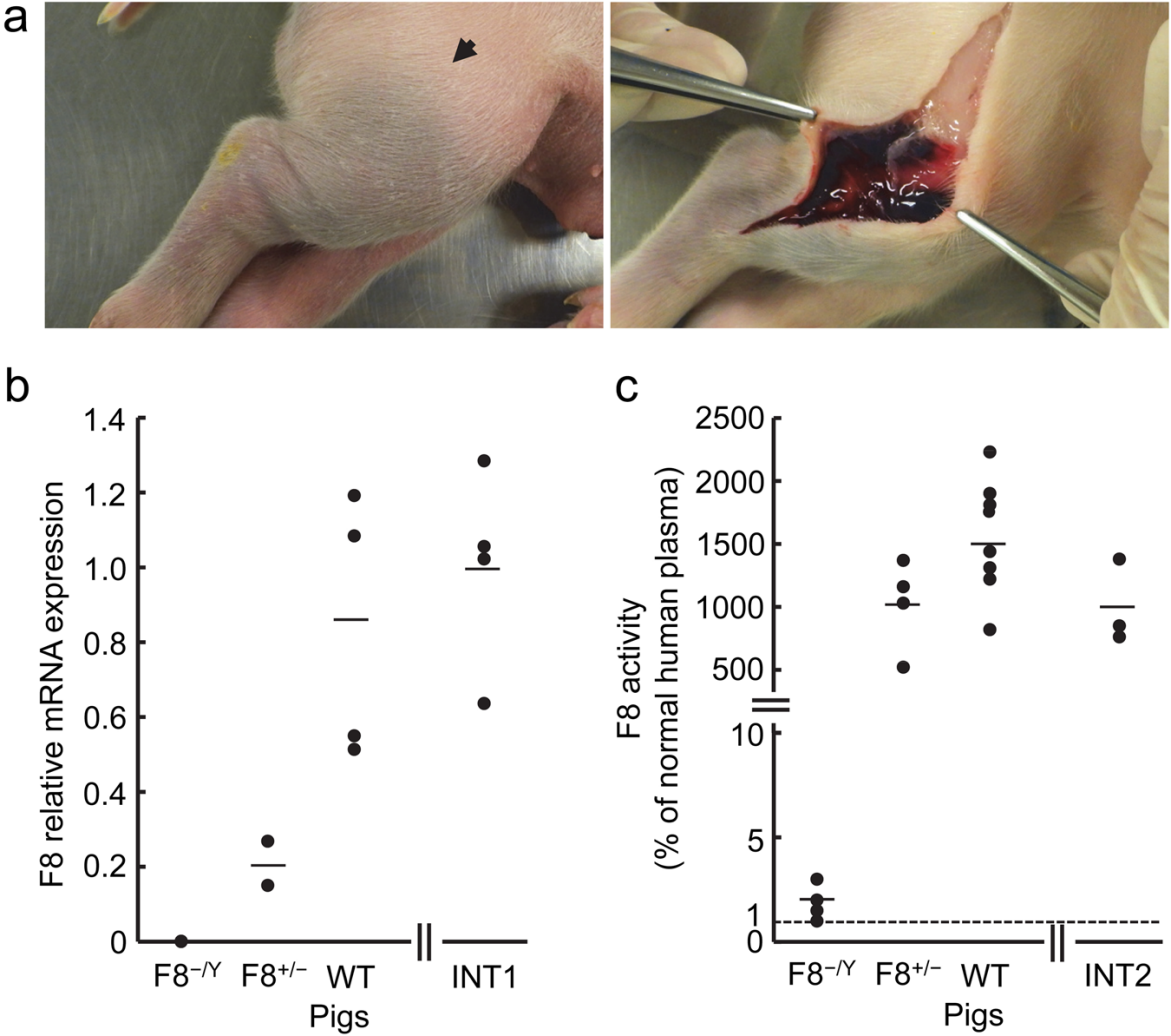
Phenotype of F2 offspring. (**a**) An example of ecchymosis in the hind limbs of F8^−/Y^ pig 1 day after birth. (b) Expression of F8 mRNA in the livers of the live-born F8^−/Y^ (n = 4), F8^+/−^ (n = 2) and wild-type (WT, F8^+/Y^, n = 4) piglets. The expression in intact neonatal piglets (2 days old) whose ancestors had no relation to the gene modification is also shown (INT1). (**c**) The F8 activity in the circulation of live-born F8^−/Y^ (n = 4), F8^+/−^ (n = 4) and wild-type (WT, F8^+/Y^ and F8^+/+^, n = 8) piglets. The activity in intact young piglets (30 days old) whose ancestors had no relation to the gene modification is also shown (INT2). The arrow in (**a**) indicates ecchymosis. Short horizontal lines in (**b**) and (**c**) indicate the mean values in each group. The dotted line in (**c**) indicates the detection limit (1%) in the assay of F8 activity.

Clinically, F8^−/Y^ piglets exhibited ecchymosis of the hind limbs or face until the day after birth (Fig. 7a). However, F8^+/−^ piglets showed no bleeding and survived. Expression of F8 mRNA was not detectable in the liver of the live-born F8^−/Y^ piglets (n = 4), whereas the expression was 0.8 ± 0.2, i.e. much higher (P < 0.01), in the wild-type (F8^+/Y^) piglets (n = 4) (Fig. 7b). Two F8^+/−^ piglets expressed a significant amount of F8 mRNA (0.15 and 0.27) (Fig. 7b). The mRNA expression was 1.0 ± 0.1 (n = 4) in the neonatal piglets (intact piglets 1, 2 days old) whose ancestors had no relation to the gene modification.

The activity of F8 in plasma of the live-born F8^−/Y^ piglets was much lower (P < 0.01), less than 3% (n = 4), as compared to the activity detected in wild-type (F8^+/Y^ and F8^+/+^) piglets (1568 ± 160%; mean ± SEM, n = 8) (Fig. 7c). In F8^+/−^ piglets, the activity was 1020 ± 181% (n = 4). The F8 activity was 1000 ± 193% (n = 3) in the young pigs (intact piglets 2, 30 days old) whose ancestors had no relation to the gene modification. There was no significant (P > 0.05) difference in F8 activity among the wild-type (F8^+/Y^ and F8^+/+^), F8^+/−^ and intact piglets 2.

## Discussion

Our institution has developed the haemophilia-A pigs using a combination of F8 gene targeting and somatic cell nuclear transfer^17^. Despite having a recessive X-linked disease, the heterozygous female cloned pigs (F8^+/−^) unexpectedly died of severe bleeding at an early age. Since the hemizygous cloned pigs (F8^−/Y^) originally exhibit a fatal bleeding phenotype^17^, conventional reproductive procedures using mature germ cells are not applicable to such cloned pigs that die quite young. In the present study, we successfully produced F8^+/−^ pigs by ICSI using xenogeneic sperm derived from the foetal F8^−/Y^ cloned pigs, and then established a strain of haemophilia A pigs using these F8^+/−^ pigs as founders. The most important findings of our study were that 1) testicular xenografting allowed generation of offspring even from foetal male pigs, and 2) the present F8^+/−^ pigs, derived from fertilized oocytes injected with xenogeneic sperm, showed no haemophilia phenotype, unlike the F8^+/−^ cloned pigs.

It has been proven by ICSI that xenogeneic sperm derived from neonatal or young donor testis have potency to generate offspring in rabbits^8^, pigs^10,19^ and primates^20^. A recent study has demonstrated completion of spermatogenesis in porcine foetal testicular xenografts^13^, although the developmental ability of xenogeneic sperm of foetal origin has remained undetermined. To our knowledge, this is the first reported study to have successfully produced live piglets by ICSI using sperm from foetal testicular xenografts. In comparison with our previous study of mice xenografted with testicular tissues obtained from wild-type foetuses at 55 or 90 days post-insemination (dpi)^13^, the present xenografts from foetal F8^−/Y^ pigs seemed to differentiate to an equal degree; the proportion of seminiferous tubule cross-sections containing elongated spermatids or sperm was 46% at graft recovery (300–369 days post-grafting), similar to the proportion in 55-dpi (44%) and 90-dpi (55%) xenografts at 360–420 days post-grafting reported previously^13^. In addition, there were no apparent differences in the serum concentrations of inhibin and testosterone between the present and previous host mice in the above periods (inhibin, 4.1 ng/ml in the present study vs 3.3–4.1 ng/ml in the previous study^13^; testosterone, 0.6 ng/ml vs 0.47–0.5 ng/ml). Since inhibin and testosterone in the serum of castrated host mice are derived from Sertoli cells^21,22^ and Leydig cells^23^, respectively, in porcine xenografts, there seemed to be no difference in the populations and functional status of these respective cell types between wild-type and F8^−/Y^ xenografts. Thus, even testicular tissue obtained from foetal gene-modified pigs can differentiate and produce functional sperm in host mice, which extends the previous finding^20^ that spermatogenesis was initiated in fresh grafted tissue obtained from a juvenile monkey transfected with the green fluorescent protein gene. In addition, this study involved cryopreservation of testicular tissue; therefore, our successful production of progeny suggests that testicular tissue from foetal gene-modified pigs is an important genetic resource worth conserving.

X-chromosome inactivation is an epigenetic process that equalizes the dosage of X-linked genes between males and females by inactivating randomly one of the two X-chromosomes present in female embryos^24–26^. This random inactivation creates mosaicism^27^ consisting of two populations of F8-producing cells such as liver sinusoidal endothelial cells^28^ in human female carriers (F8^+/−^) for haemophilia; one cell population has an activated intact X-chromosome and can produce F8, while the other has an activated X-chromosome with F8 gene mutations and cannot produce F8. This situation is enough to ensure normal blood clotting, and therefore many female carriers are asymptomatic^15^. However, heterozygous women may have an extremely skewed X-inactivation pattern; one X-chromosome may be more extensively inactivated than the other^29^, resulting in a paucity of circulating F8 activity and severe bleeding^30–32^. Like heterozygous women with extremely skewed X-inactivation, our F8^+/−^ cloned pigs also showed severe bleeding. The precise reasons for this phenomenon have not been identified. However, aberrant expression of X-linked genes has been reported in cloned pigs^33,34^, as wells as cloned mice^35,36^ and cattle^37^. It seems likely that insufficient reprogramming of X-inactivation may occur in F8^+/−^ cloned embryos, eventually leading to an extreme reduction in the proportion of F8-producing cells. Abnormal X-inactivation also probably impairs lymphoid development in female immunodeficient pigs with heterozygous disruption of their X-linked interleukin-2 receptor gamma genes by nuclear transfer cloning^38,39^.

The present F8^+/−^ pigs, produced by ICSI using sperm derived from foetal F8^−/Y^ cloned pigs, were asymptomatic, unlike the F8^+/−^ cloned pigs. Similarly, other phenotypic abnormalities seen in cloned mice^18,40^ and pigs^38^ were not transmitted to the progeny. Grafted tissues from F8^−/Y^ foetuses produce spermatozoa bearing an X-chromosome with the disrupted F8 gene, and those bearing a Y-chromosome in host mice. By injection of the sperm into individual wild-type oocytes (F8^+^), the genotype of F_1_ females became heterozygous (F8^+/−^) and an F_1_ male wild type (F8^+/Y^) was expected. These F8^+/−^ pigs produced a significant amount of F8 protein, and the F8^+/−^ pigs in the F_2_ generation similarly did so. These results strongly suggest that the causes of the bleeding phenotype in the F8^+/−^ cloned pigs were not genetic mutations but epigenetic modifications, as the phenotype disappeared in the progeny of the F8^+/−^ cloned pigs.

Successful delivery of F_2_ offspring by the F_1_ heterozygous pigs indicates that female pigs produced using xenogeneic sperm of foetal origin have normal reproductive ability, as is the case for pigs produced using sperm from neonatal xenografts^12,41^. The proportions of the respective genotypes in the F_2_ offspring were F8^−/Y^: 29.4%, F8^+/−^: 23.5%, F8^+/Y^: 23.5% and F8^+/+^: 23.5%, strongly suggesting that the disruption of the F8 gene is still conserved on the X-chromosome. The F8^−/Y^ pigs in the F_2_ generation did not express F8 mRNA in their liver cells and showed spontaneous bleeding due to lack of F8 activity, thus indicating that the severe haemophilia phenotype seen in the F8^−/Y^ cloned pigs^17^ had been transferred to their progeny. On the other hand, F8^+/−^ pigs were asymptomatic because of the presence of F8 activity in the circulation. The activity varied (521% to 1370%) and did not differ from that in wild-type pigs; these findings are consistent with a study of heterozygous women in which the subjects exhibited a wide range of circulating F8 levels^32^. Although we assessed the mRNA expression of the F8 gene in only two F8^+/−^ pigs because of their high value as founders, they expressed a significant amount of F8 mRNA in the liver. Thus, we have succeeded in establishing a strain of haemophilia-A pigs using F8^+/−^ pigs derived from foetal F8^−/Y^ cloned pigs as founders, allowing continuous production of F8^−/Y^ pigs.

A convenient murine model of haemophilia-A has already been developed by targeted disruption of the mouse F8 gene^42,43^. However, these knockout mice rarely exhibit spontaneous bleeding coupled with long-term survival without treatment of F8 variants: the difference in phenotype between F8-distrupted mice and human patients with severe haemophilia-A would limit the preclinical usefulness of this model. In addition, as mice are short-lived, the effects of treatment cannot be monitored over a long period. Our F_2_ progeny (F8^−/Y^) showed spontaneous bleeding, similarly to human patients with severe haemophilia^15^. Since pigs are basically long-lived animals, use of the porcine haemophilia-A strain will facilitate longitudinal assessment of the efficacy of new F8 variants and gene therapy using adeno-associated virus^44^.

## Methods

### Experimental animals

All experiments were performed in accordance with protocols for the experiments that were approved by the Animal Care Committee (#H18–008–02, #H18–038, #H28-P07 and H28-002) and Gene Recombination Experiment Safety Committee (#1444772-2 and #500035-30) of the Institute of Agrobiological Sciences, National Agriculture and Food Research Organization (NARO), Tsukuba, Japan. Crossbreed pigs (Landrace × Large White × Duroc) used in this study were produced and reared according to the Japanese Feeding Standard for Swine (2005) at the Institute of Livestock and Grassland Science of NARO. Male nude mice (Crlj:CD1-Foxn1^nu^) 5–6 weeks old, purchased from Charles River Japan (Yokohama), were kept in an environmentally controlled room maintained at a temperature at 24 °C and 50% humidity, and illuminated daily from 05:00 to 19:00.

### Chemicals

All chemicals were purchased from the Sigma-Aldrich Corporation (St. Louis, MO, USA), unless otherwise indicated.

### Production of F8^**−**/Y^ pigs

F8^−/Y^ pigs (F_0_ generation) were produced by the cloning technique described previously^17,38,45^. Briefly, porcine fibroblasts were isolated from male foetuses approximately 60 days after artificial insemination. The construct of the targeting vector was the exon 16 DNA fragment with a deletion of 11 nucleotides containing an inserted PGK-Neo cassette flanking a 5′ arm of 3.2 kb and a 3′ arm of 4.1 kb (each of genomic F8 DNA)^17^. The HSV-TK cassette was located on the 5′ end of the 5′ arm. Approximately 1 × 10^7^ cells were transfected with 5 nM targeting vector by electroporation (Gene Pulser II; Bio-Rad, Hercules, CA, USA) at 278 V/cm and 950 μF. Electroporated cells were then incubated with G418 (800 µg/ml, Nakarai Tesque, Kyoto, Japan) for 10 days, and drug-resistant cells were selected and subjected to further incubation. After reaching confluence, half of the confluent cells were subjected to PCR analysis to identify the targeting event: the remainder were stored in liquid nitrogen until use for nuclear transfer. Nuclei of the targeted cells were directly injected into the cytoplasm of *in vitro*-matured oocytes after enucleation using a piezo-actuated micromanipulator (PMAS-CT150, Prime Tech, Tsuchiura, Japan). The oocytes were then stimulated with a direct current pulse of 1.5 kV/cm for 100 μs using a somatic hybridizer (SSH-10; Shimadzu, Kyoto, Japan). These reconstituted oocytes were incubated until they reached the 2- to 8-cell embryo stage. Cleaved embryos (150–300 embryos) were surgically transferred to both oviducts of individual estrus-synchronized recipient gilts (n = 25).

### Collection and vitrification of foetal testicular tissue

Foetal testes were obtained from three F8^−/Y^ cloned pigs between 69 and 83 days after embryo transfer. Simultaneously, portions of auricles and liver were collected for analysis of genotype by PCR and Southern blotting and expression of F8 mRNA by quantitative RT-PCR. Foetal testes were minced into fragments measuring approximately 1.5 × 1.5 × 1.5 mm^13^ and the fragments were vitrified according to the method described by Dinnyes *et al*.^46^ and Somfai *et al*.^47^, with some modifications^10,13^. In brief, fragments were washed in base solution at room temperature (BS: IVC-PyrLac solution^48^ supplemented with 20 mM HEPES (Dojindo, Kumamoto, Japan)), and then transferred to 500 μl of equilibration solution (BS supplemented with 4% ethylene glycol (EG)) and incubated for 15 min. The equilibrated fragments were immersed in 500 μl of vitrification solution (BS supplemented with 35% EG, 5% polyvinyl pyrrolidone (Mr 40000) and 0.3 M trehalose) for 10 min. Each fragment was dropped onto an aluminum foil boat partially immersed in liquid nitrogen to form a micro-droplet. Finally, the vitrified droplets were stored in liquid nitrogen.

### Xenografting of foetal testicular tissue after warming

After confirmation of the presence of the targeted allele in foetal donors by PCR and Southern blotting, vitrified droplets containing testicular fragments were transferred to a warming solution (BS supplemented with 0.4 M trehalose) at 37 °C for 2 min^10,13^. Fragments were consecutively transferred for 2-min periods to BS supplemented with 0.2 M, 0.1 M, and 0.05 M trehalose to remove cryoprotectants. Approximately 15 fragments were inserted under the back skin of individual mice (n = 7), immediately after castration under anesthesia with pentobarbital sodium (Somnopentyl; Kyoritsu Pharmaceuticals, Tokyo, Japan)^10,13,49^.

### Sperm recovery and ICSI

Host mice were euthanatized for collection of blood samples between 300 and 369 days after grafting (n = 4). Immediately after blood sampling, grafts were recovered from each mouse in collection medium (Dulbecco’s PBS; Nissui, Tokyo, Japan, supplemented with 5 mg/ml BSA). Three pieces were excised from the different larger grafts and fixed in Bouin’s solution for histology. The remaining portions were cut into small pieces in the collection medium for recovery of sperm. The tissue suspension was centrifuged for 10 min at 600 × *g*, and the supernatant was discarded. After washing with the collection medium 3 times^10,13,19^, the pellet was resuspended in a small volume of collection medium and maintained at room temperature until use for ICSI.

ICSI was performed in accordance with our previous studies^10,13,19,50–53^. Beforehand, cumulus-oocyte complexes (COCs) collected from wild-type gilts were subjected to *in vitro* maturation for 44–46 h^48^. Expanded COCs were denuded of their cumulus cells mechanically after treatment with 150 IU/ml hyaluronidase: oocytes showing extrusion of the first polar body were harvested as mature oocytes. A morphologically normal single sperm was aspirated tail first and injected into the ooplasm of the mature oocyte using a piezo-actuated micromanipulator (Prime Tech). The oocyte was then stimulated with a direct current pulse of 1.5 kV/cm for 20 μs using a somatic hybridizer. Parthenogenetic oocytes for assistance of pregnancy^54,55^ were generated by electrostimulation with a direct pulse of 2.2 kV/cm for 30 μs.

### Production of F_1_ pigs

Mixtures of 70–90 sperm-injected oocytes and approximately 30 parthenogenetic oocytes were surgically transferred to both oviducts of individual estrus-synchronized recipients (n = 4). Farrowing was synchronized using a prostaglandin F2α analog (cloprostenol) (Nagase Medicals, Itami, Japan) at 113–116 days after transfer of sperm-injected oocytes. Piglets (F_1_ generation) were conventionally nursed and observed daily to examine the presence of ecchymosis. At 3 months after birth, auricles and blood were collected from F_1_ pigs for genotype analysis and plasma F8 activity measurement (n = 3).

### Production of F_2_ pigs

After confirmation of genotype, F_1_-generation F8^+/−^ pigs were artificially inseminated with fresh semen from wild-type (F8^+/Y^) boars (Landrace or Large white). Farrowed male F_2_ piglets, theoretically consisting of F8^−/Y^ and F8 ^+/Y^ genotypes, were euthanatized by the day after birth to avoid sudden death due to bleeding (n = 9). Blood, auricles and liver were collected for analyses of genotype and phenotype. Female F_2_ piglets, probably consisting of F8^+/−^ and F8 ^+/+^, were reared conventionally. Portions of the auricles and blood were then collected within 30 days after birth (n = 6), except for two F8^+/−^ female piglets that were euthanatized by the day after birth for collection of liver tissue for measurement of F8 mRNA expression.

### PCR analysis

PCR analysis was performed on electroporated cells, foetal F_0_ clones, and F_1_ and F_2_ progeny. Electroporated cells were digested with proteinase K (Nakarai Tesque) solution and the mixture was used as a template: from ear tissues of foetuses and piglets, genomic DNA was prepared as a template using a GenoPlus Genomic DNA Extraction Miniprep System (Viogene, Taipei, Taiwan)^17,38,56^. In both cases, PCR amplification was performed with LA Taq Hot Start Version (Takara Bio, Otsu, Japan), using the primers Neo sF: 5′-CGCCTTCTTGACGAGTTCTTCTG-3′ and Exon 22 sR: 5′-TAAGGTGCCCGTGGAATTCCCTC-3′^17^ (defined as Neo-Ex 22 PCR) (Fig. 2). Thermal cycling parameters were: 5 min at 94 °C, followed by 35 cycles of 30 s at 94 °C, then 8 min at 68 °C, followed by a final step of 7 min at 68 °C. This amplification yields a mutant-allele-specific product of 6.0 kb.

### Southern blotting analysis

Genomic DNA was extracted from ear tissue obtained from foetal F_0_ clones, F_1_ and F_2_ progeny by the phenol/chloroform method. Ten micrograms of genomic DNA digested with Sph I or Sac I was subjected to agarose gel electrophoresis followed by blotting onto nylon membranes (Roche, Basel, Switzerland) as described previously^38,56^. Digoxigenin-labeled 5′ and 3′ probes generated by PCR (497 bp from exon 14 of genomic F8 DNA and 469 bp from exon 22, respectively)^17^ were applied to Sac I- and Sph I-digested DNA, respectively (Fig. 2). The signals were visualized using CSPD (Roche).

### Quantitative RT-PCR

Total RNA was isolated from liver tissue obtained from foetal F_0_ clones and F_2_ progeny using Sepazol (Nakarai Tesque) and converted to first-strand cDNA with a PrimeScript RT reagent kit (TaKaRa Bio)^38,57^. Real-time quantitative RT-PCR of the transcripts of interest was carried out with a LightCycler instrument (Roche). PCR amplification was performed with SYBR premix Ex Taq II (Takara Bio) using primers Exon 19 F: 5′-TCAGAGCGGAAGTTGAAGAC-3′ and Exon 20 R: 5′-ACGAAGTTGTGTCGAGGTTC-3′ specific for the porcine F8 gene. The relative degree of F8 gene expression was normalized against expression of the β-actin gene using a pair of primers (ACTB F: 5′-AGGTCATCACCATCGGCAAC-3′ and ACTB R: 5′-ATCTCCTTCTGCATCCTGTC-3′). The cycling conditions were 95 °C for 3 min, followed by 60 cycles at 95 °C for 5 s, 55 °C or 58 °C for 30 s each, and 72 °C for 30 s.

### Histology

Grafts obtained from host mice were cut into sections 6 μm thick and stained with hematoxylin and eosin. The seminiferous cord or tubule cross-sections were then sorted into the following categories, as described previously^10,13,49^: (1) no germ cells present (cord or tubule cross-sections showing Sertoli cells only); (2) gonocytes or spermatogonia present; (3) spermatocytes present as the most advanced germ cells; (4) round spermatids present as the most advanced germ cells; (5) elongated spermatids present as the most advanced germ cells; and (6) mature sperm (spermatozoa) present in the lumina of the tubules in which spermatids coexisted. All seminiferous cord or tubule cross-sections observed in one section of each testicular tissue were categorized. The data obtained from 3 fragments from each mouse were pooled and the percentages calculated. Mean (±SEM) percentages per mouse were calculated using the data obtained from 4 host mice.

### Measurement of total inhibin and testosterone

Total inhibin in the sera of the host mice was determined by a competitive fluoroimmunoassay (FIA) using europium (Eu)-labeled inhibin A as a probe^21,58,59^. For the FIA, anti-bovine inhibin serum (TNDH-1)^60^ was used as a primary antibody. Bovine 32-kDa inhibin A was used for Eu-labeling^21,59^ and as a reference standard^21,58,59^ (the anti-inhibin serum was provided by Dr. K. Taya, Tokyo University of Agriculture and Technology, Fuchu, Japan; bovine 32-kDa inhibin was purchased from Protein Purity Co, Isezaki, Japan). The detection limit of the FIA was 0.078 ng/ml.

The concentrations of testosterone were determined with a commercial competitive FIA kit (DELFIA testosterone kits, PerkinElmer Japan, Yokohama, Japan). Testosterone was extracted from the serum with 2 ml of ether before being applied to the kit. The detection limit of the FIA was 1.0 pg/ml.

### Measurement of F8 activity

Activity of F8 in plasma of piglets was measured at a clinical laboratory (Nagahama Life Science Laboratory, Shiga, Japan) using a clotting time assay kit (Cosmo Bio, Tokyo, Japan). Normal human plasma was used as a reference standard. The F8 activity was expressed as a percentage of the F8 activity in normal human plasma.

### Statistical analysis

Expression of F8 mRNA and F8 activity in plasma was sorted according to genotype and analysed by one-way ANOVA. The differences were then tested by Tukey’s test. The General Linear Models of Statistical Analysis Systems, ver 9.2 (SAS Inc., Cary, NC, USA) was used for these analyses. Differences at P < 0.05 were considered significant.

### Data availability

The datasets generated during and/or analysed during the current study are available from the corresponding author on reasonable request.

## Electronic supplementary material

Supplementary information
