# Supplementary material for: Establishment of a strain of haemophilia-A pigs by xenografting of foetal testicular tissue from neonatally moribund cloned pigs
Source: Sci Rep. 2017 Dec 5;7:17026. doi: 10.1038/s41598-017-17017-6 (PMC5717049; doi:10.1038/s41598-017-17017-6)
Supplement: Supplementary file 1 — Supplementary information [file 41598_2017_17017_MOESM1_ESM.pdf]

Supplementary Information

Establishment of a strain of haemophilia-A pigs by xenografting of foetal testicular tissue from neonatally moribund cloned pigs

Hiroyuki Kaneko[1], Kazuhiro Kikuchi[1,2], Michiko Nakai[1,†], Daiichiro Fuchimoto[1], Shunichi Suzuki[1], Shoichiro Sembon[1,†], Junko Noguchi[1], Akira Onishi[3]

[1]Institute of Agrobiological Sciences, National Agriculture and Food Research Organization (NARO), Tsukuba, Ibaraki, 305-8602, Japan. [2]The United Graduate School of Veterinary Science, Yamaguchi University, Yoshida, Yamaguchi, 753-8515, Japan. [3]Nihon University, College of Bioresource Sciences, Fujisawa, Kanagawa, 252-0880, Japan. †Present address: NARO, Tsukuba, Ibaraki, 305-8517, Japan.

Supplementary Methods S1

Production of female F8-targeted pigs. Female F8-targeted pigs were produced by nuclear transfer cloning from female foetal fibroblasts after disruption of the X-linked coagulation factor VIII (F8) gene, according to the methods used for production of $F8^{-/Y}$ cloned pigs (see Methods in the main text).

Genotype analysis of female F8-targeted cloned pigs by PCR. Genomic DNA was prepared as a template from ear tissues of piglets using a GenoPlus Genomic DNA Extraction Miniprep System (Viogene, Taipei, Taiwan)[17, 38, 56]. The targeted F8 alleles were amplified by two sets of primer pairs for exon 14 and PGK-Neo (Ex14-Neo PCR), and for PGK-Neo and exon 22 (Neo-Ex22 PCR, see the figure below). The primer set used for Ex14-Neo PCR was Exon 14 sF: 5′-GCTTTATTAACTGAGAATAGGGCATCTGC-3′ and Neo sR: 5′-CTTCCCGCTTCAGTGACAACGTCGAGCACAG-3′, and that for Neo-Ex 22 PCR was Neo sF: 5′-CGCCTTCTTGACGAGTTCTTCTG-3′ and Exon 22 sR: 5′-TAAGGTGCCCGTGGAATTCCCTC-3′. The predicted product sizes were 7.7 and 6.0 kb, respectively. The corresponding wild-type alleles were also amplified by two sets of primers for exons 14 and 16 (Ex14-16 PCR) and for exons 16 and 22 (Ex16-22 PCR). The primer set used for Ex14-16 PCR was Exon 14 sF: 5′-GCTTTATTAACTGAGAATAGGGCATCTGC-3′ and Exon 16 sR: 5′-CTCCTGATCATCCGGATAAG-3′, and that for Ex16-22 PCR was Exon 16 sF: 5′-CGAGCCTTATTTCTTATCCG-3′ and Exon 22 sR: 5′-TAAGGTGCCCGTGGAATTCCCTC-3′. The predicted product sizes were 7.0 and 5.5 kb, respectively. The 3′ ends of Exon 16 sF and sR were located in an 11-nucelotides stretch deleted by targeting events, so that these primers yielded no amplicons from the F8-targeted allele.

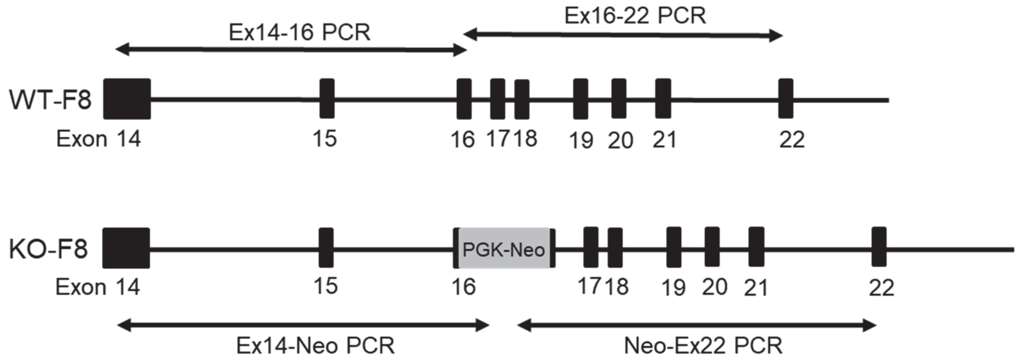

Figure. Diagram of wild-type porcine F8 (WT-F8) and the targeted F8 (KO-F8) genes. Positions of PCR primers and amplified DNA fragments are indicated by arrows.

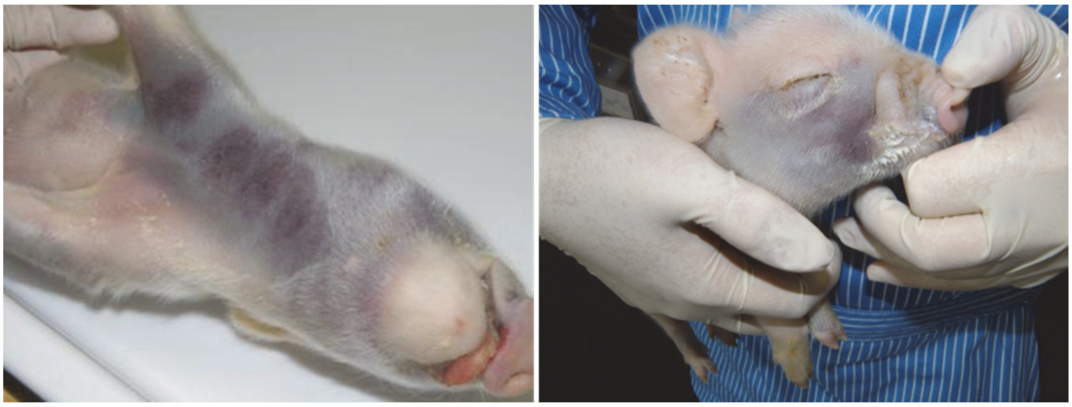

Supplementary Figure S1. The bleeding phenotype in female F8-targeted cloned pigs. Two individual images of female F8-targeted cloned pigs, which died within 3 days after birth, showing ecchymosis from the cheek to the neck.

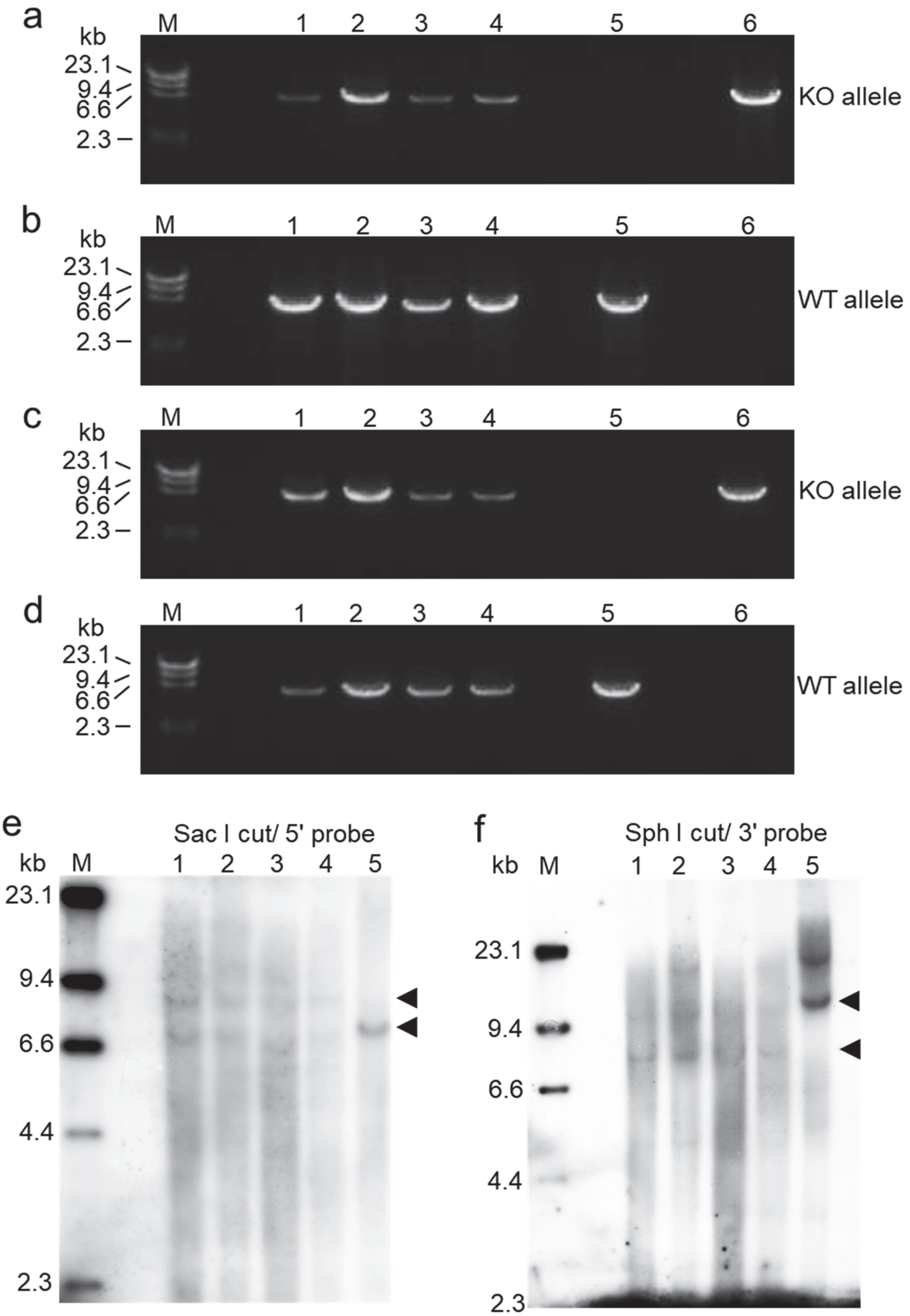

Supplementary Figure S2. Genotype analysis of female F8-targeted cloned pigs by PCR and Southern blotting. Amplification of (a) targeted (KO) and (b) wild-type (WT) F8 alleles by Ex

14-Neo PCR (a) and Ex 14-16 PCR (b) (see Supplementary Methods S1). Amplification of (c) targeted (KO) and (d) wild-type (WT) F8 alleles by Neo-Ex 22 PCR (c) and Ex 16-22 PCR (d) (see Supplementary Methods S1). From (a) to (d), Lanes 1 to 4; female F8-targeted piglets, lane 5; wild-type control, lane 6; positive control, a male $F8^{-/Y}$ pig produced in the previous study[17]. Southern blotting of (e) Sac I- and (f) Sph I-digested genomic DNA (see Methods in the main text). Lanes 1 to 4; female F8-targeted piglets, lane 5; wild-type control. Arrowheads indicate hybridized signals. M indicates molecular weight marker (λ-Hind III). Note that female F8-targeted cloned pigs have one copy of the targeted F8 allele and one copy of the wild-type F8 allele.

Fig. 3b

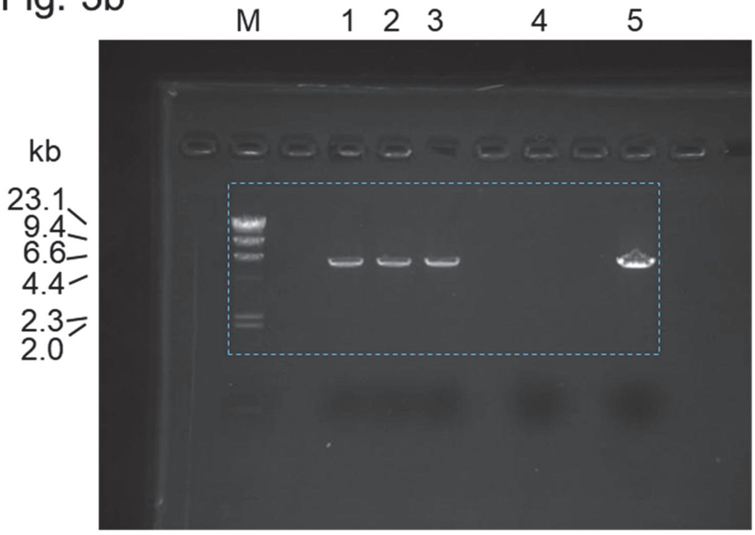

Fig. 3c

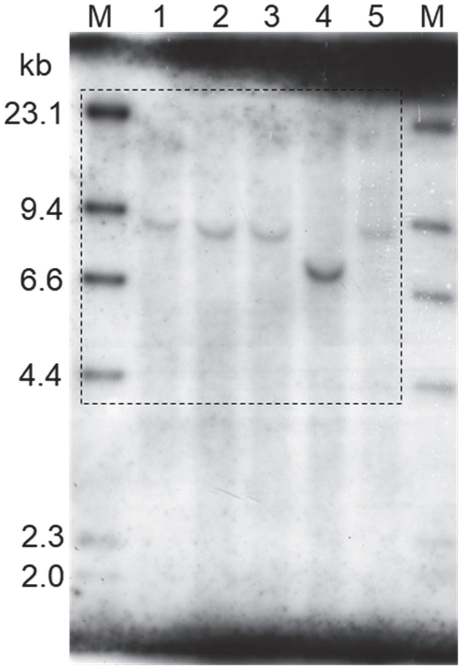

Fig. 3d

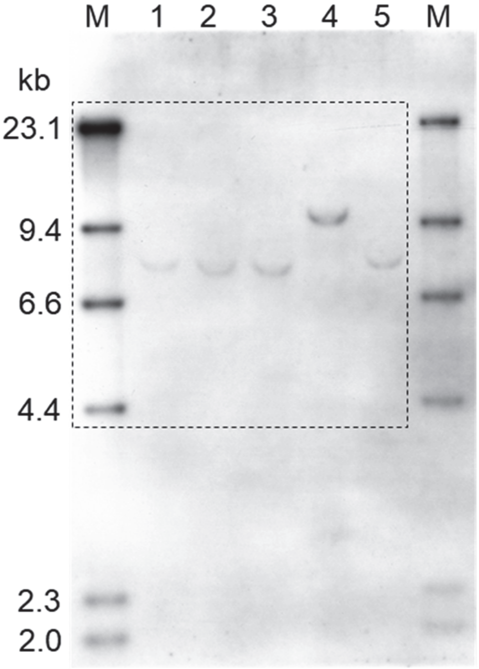

Supplementary Figure S3. Full-length images of PCR and Southern blotting of Figs. 3b-d.

Dotted areas were cropped and used for the figure.

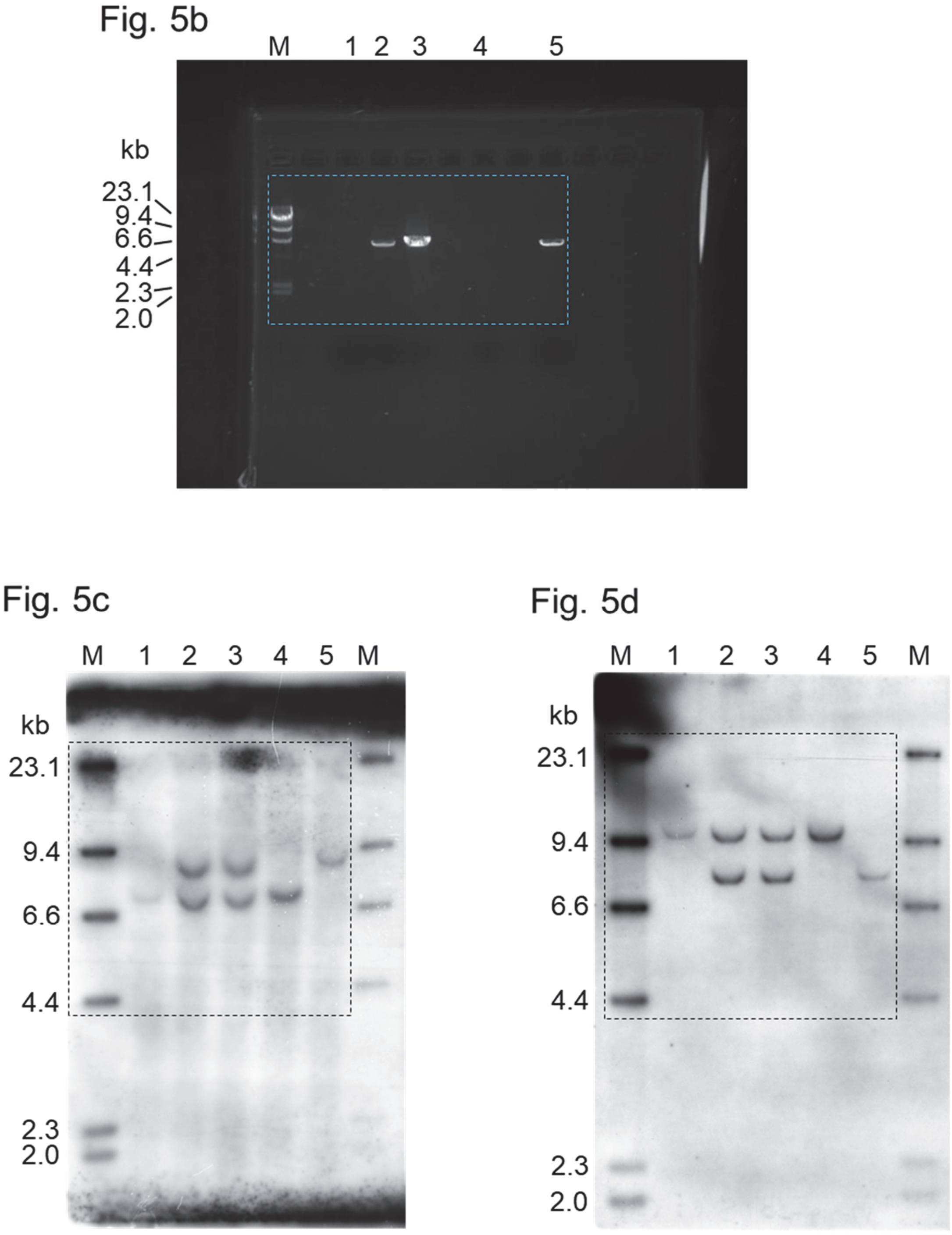

Supplementary Figure S4. Full-length images of PCR and Southern blotting of Figs. 5b-d.

Dotted areas were cropped and used for the figure.

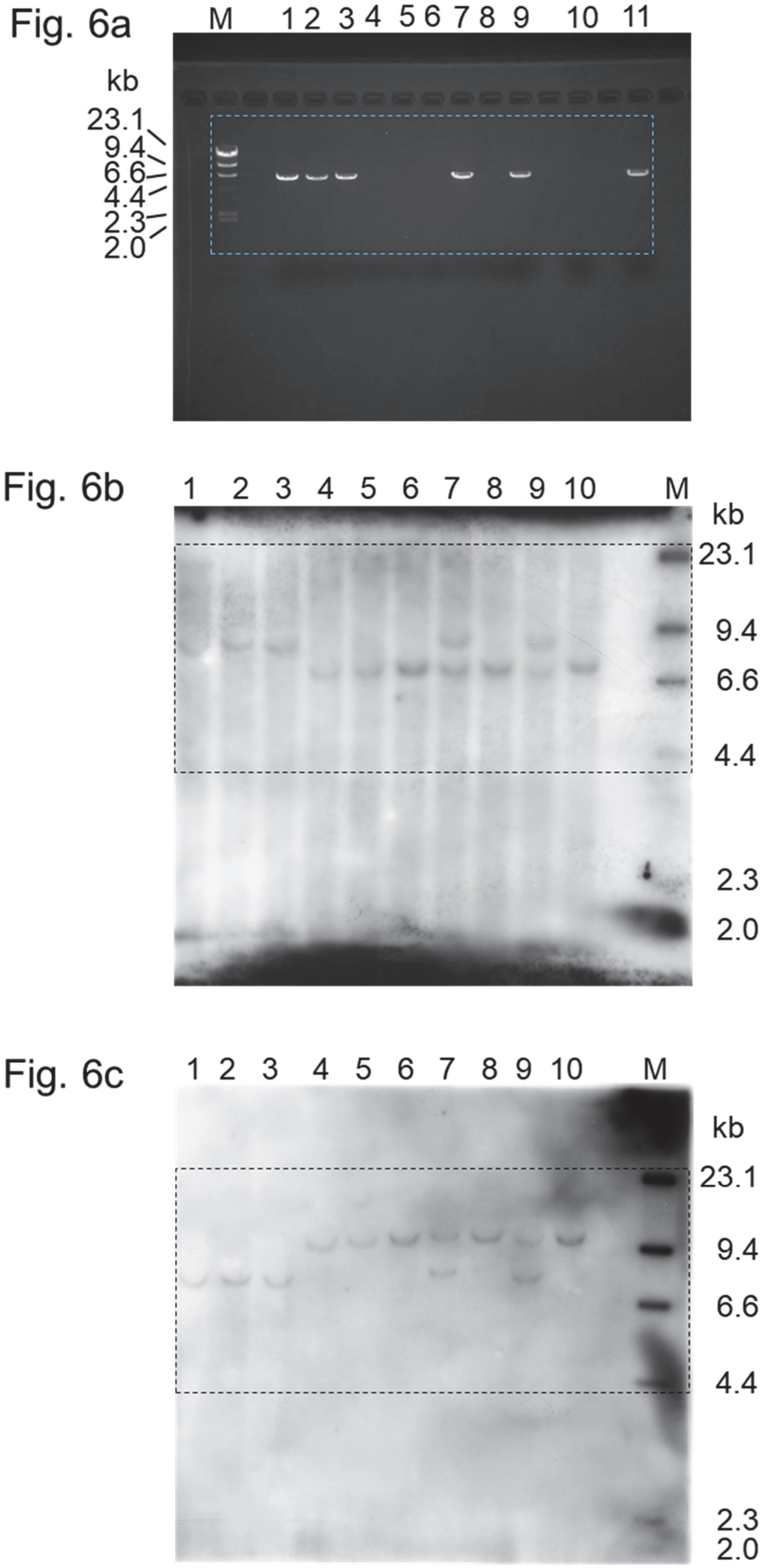

Supplementary Figure S5. Full-length images of PCR and Southern blotting of Figs. 6a-c.

Dotted areas were cropped and used for the figure.
